# Astroviruses in bats, Madagascar

**DOI:** 10.1038/emi.2017.47

**Published:** 2017-06-21

**Authors:** Camille Lebarbenchon, Beza Ramasindrazana, Léa Joffrin, Sandra Bos, Erwan Lagadec, Gildas Le Minter, Yann Gomard, Pablo Tortosa, David A Wilkinson, Steven M Goodman, Patrick Mavingui

**Affiliations:** 1 University of Reunion Island, UMR PIMIT (Processus Infectieux en Milieu Insulaire Tropical), INSERM 1187, CNRS 9192, IRD 249. GIP CYROI, 2 Rue Maxime Rivière, 97490 Sainte-Clotilde, La Réunion, France; 2 Association Vahatra, BP 3972, Antananarivo 101, Madagascar; 3 Field Museum of Natural History, 1400 South Lake Shore Drive, Chicago, IL 60605, USA

**Dear Editor,**

Astroviruses (AstVs) (family *Astroviridae*) are positive-sense, single-stranded RNA viruses that infect a large diversity of mammalian and avian species.^1^ In humans, eight serotypes have been described worldwide, accounting for 2%–9% of all acute non-bacterial gastroenteritis cases in children.^2^ AstVs have been detected from over 80 non-human host species, including a large diversity of bat species in Asia, Europe and Africa,^3^ and recent phylogenetic analyses suggest that the long-term evolution of AstVs is determined by frequent cross-species transmission events.^4, 5^ During the past decade, numerous AstVs with potential zoonotic transmission have been described, highlighting the need for improved knowledge on their biology to prevent future health threats.^2^

Bats are recognized as a major reservoir of infectious agents. Madagascar shelters over 46 bat species, of which nearly 80% are endemic, occupying different types of day roosts, including natural and synanthropic sites. Recent investigations have shown that the compositions of Malagasy bat species assemblages are correlated with factors associated with the diversity and transmission of infectious agents, such as paramyxoviruses and *Leptospira*.^6, 7^ The detection of new Betacoronaviruses in endemic Malagasy fruit bats also underlined the urgent need to better understand bat-associated infectious agents, and thus assess the potential for spillover to human populations in this major biodiversity hotspot.^8^

Herein, Malagasy bats were sampled for the detection of AstVs in the context of a multilevel research program examining the systematics and biogeography of these animals and their associated infectious agents. Bats were trapped at two locations (Ambohitantely and Anjohibe) using mist nets, harp traps and butterfly nets. In Anjohibe, several different species were trapped in a large cave network, whereas in Ambohitantely, two species were sampled at different sites: *Myotis goudoti* in a cave roost and *Mormopterus jugularis* in a public school. Rectal and buccal swabs were obtained using sterile rayon-tipped applicators (Puritan, Guilford, ME, USA). Pooled swabs from each individual bat (one rectal+one buccal swab) were placed in the same tube containing 1.5 mL of brain heart infusion medium (Conda, Madrid, Spain) supplemented with penicillin G (1000 units/mL), streptomycin (1 mg/mL), kanamycin (0.5 mg/mL), gentamicin (0.25 mg/mL) and amphotericin B (0.025 mg/mL), immediately frozen in liquid nitrogen, and subsequently transferred and stored in a −80 °C freezer.

Samples were vortexed and centrifuged at 1500*g* for 15 min. RNA extraction was performed using the QIAamp Viral RNA Mini Kit (QIAGEN, Valencia, CA, USA). Reverse transcription was performed on 10 μL of RNA using the ProtoScript II Reverse Transcriptase and Random Primer 6 (New England BioLabs, Ipswich, MA, USA) under the following thermal conditions: 70 °C for 5 min, 25 °C for 10 min, 42 °C for 50 min and 65 °C for 20 min. cDNAs were tested for the presence of the AstV RNA-dependent RNA-polymerase (RdRp) gene using a pan-AstV semi-nested polymerase chain reaction (PCR) assay.^9^ PCRs were performed with the GoTaq G2 Hot Start Green Master Mix (Promega, Madison, WI, USA) in an Applied Biosystems 2720 Thermal Cycler (Thermo Fisher Scientific, Waltham, MA, USA). After electrophoresis on a 1.5% agarose gel stained with 2% GelRed (Biotium, Hayward, CA, USA), PCR products of the expected size were submitted for direct Sanger sequencing (Genoscreen, Lille, France).

Overall, 40 of 178 bats tested positive for the presence of AstV RdRp (mean detection rate±95% confidence interval: 22.5%±6.13%). This detection rate was consistent with rates reported in other studies using the same PCR assay.^9, 10^ Six of the nine bat species tested positive (Supplementary Table S1), with significant variation between species (*χ*^2^=63.3, *P*<0.001). A difference in the mean detection rates between the Ambohitantely (1.96%±3.81%) and the Anjohibe (30.7%±8.02%) sites was also observed (*χ*^2^=23.2, *P*<0.001). This result may be affected by a sampling bias between the two sites (51 bats sampled in Ambohitantely versus 127 in Anjohibe) or may reflect species- and site-related variation in AstV infection. Repeated sampling of the same bat populations would be needed to examine potential seasonal variations in virus transmission between hosts and locations.

A Bayesian Markov Chain Monte Carlo coalescent analysis was performed with 31 sequences obtained in this study (GenBank accession numbers KY575644–KY575674) and 83 reference AstV RdRp partial nucleotide sequences. Sequences (395 bp) were aligned using CLC Sequence Viewer version 7.7.1 (CLC Bio, Aarhus, Denmark). The coalescent analysis was performed with the program BEAST 1.8.4,^11^ with the general time-reversible evolutionary model, an estimation of the proportion of invariable sites (I) and of the nucleotide heterogeneity of substitution rates (α), as selected by Model Generator 0.85.^12^ A strict molecular clock and a constant population size were selected. The analysis was performed with a chain length of 60 million generations sampled every 1000 iterations; the first 10% of trees were discarded as burn-in.

Analysis of the 31 RdRp partial sequences of AstVs obtained from Malagasy bats revealed high genetic diversity (pairwise distance up to 38%). High sequence diversity is commonly described in bat AstVs,^3^ with studies reporting potentially novel AstV species in these hosts.^9, 13, 14^ The coalescent analysis provided further evidence of this considerable diversity and the low degree of host restriction (Figure 1). For instance, the two viruses detected in *Rousettus madagascariensis* were more genetically related to AstVs found in *Miniopterus griveaudi* and in *Triaenops menamena* than to each other. The close proximity of bat roosting sites in the Anjohibe cave network may favor virus transmission between species and, more broadly, the infection of a large diversity of hosts sharing these habitats. The sequence detected in *Myotis goudoti* at Ambohitantely was closely related to one of the two sequences detected in the same species at Anjohibe, further suggesting that AstVs may easily spread within bat populations. Low levels of host restriction and limited phylogenetic clustering between viruses detected at distant locations have also been reported in other studies in Asia, Europe and Africa,^3^ although the ecological drivers of AstV epidemiology and evolution remain to be determined.^4^

This report suggests that Malagasy bats may act as reservoirs of a large diversity of AstVs. Further epidemiological work is necessary to identify hosts and ecological factors involved in AstV infection among bats. Additional sequencing may also provide important information on the origin and evolutionary history of these viruses. AstVs are important agents of acute gastroenteritis among children <24 months of age on Madagascar, where simultaneous circulation of multiple genotypes has been reported.^15^ Comprehensive source-attributed case–control studies will be necessary to assess the risk of zoonotic transmission of bat-origin AstVs.

## Figures and Tables

**Figure 1:**
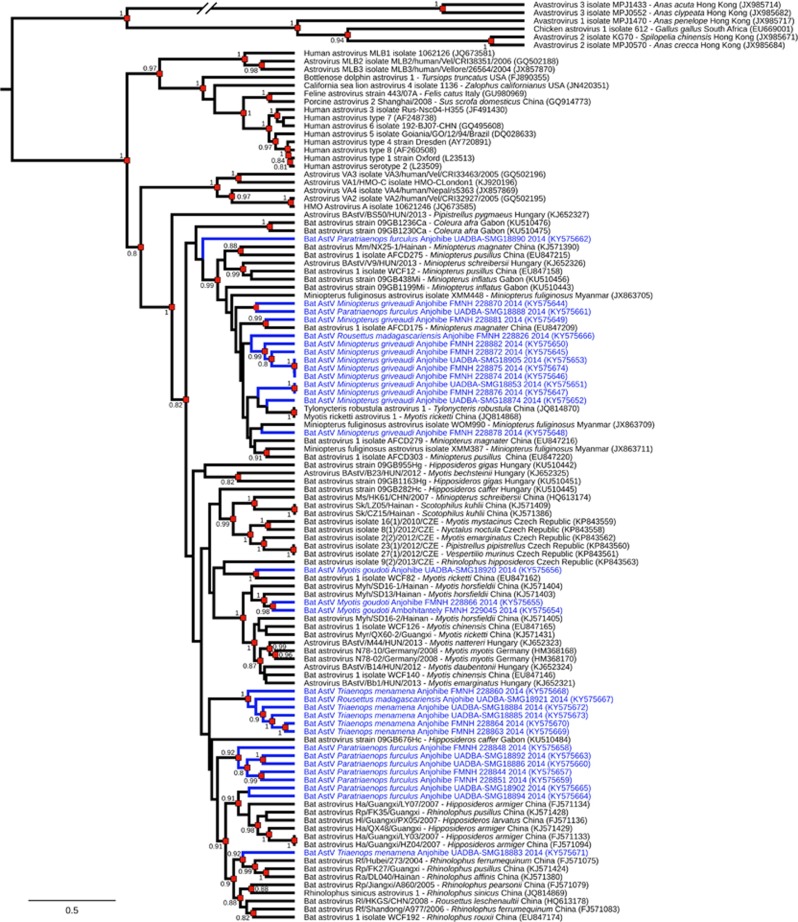
Maximum clade credibility tree from 114 Astrovirus (AstV) RNA-dependent RNA-polymerase partial nucleotide sequences (395 bp). Blue branches indicate bat AstVs detected on Madagascar. Sequence accession numbers are indicated in parentheses, and virus origin (host bat species and sampling location) is detailed for animal-origin AstVs. Posterior probabilities are reported when >0.8 (red squares). Sequences derived from this study are indicated in blue.
