# Supplementary material for: Astroviruses in bats, Madagascar
Source: Emerg Microbes Infect. 2017 Jun 21;6(6):e58–. doi: 10.1038/emi.2017.47 (PMC5520320; doi:10.1038/emi.2017.47)
Supplement: Supplementary Table S1 [file emi201747x1.pdf]

Supplementary Table 1. Location, collection date, species and number of bats sampled and tested for the presence of Astroviruses on Madagascar.

**Location**	**Date**	**Family**	**Species**	**N tested**	**N positive**
Ambohitantely	Oct. 2014	Molossidae	*Mormopterus jugularis*	40	0
		Vespertilionidae	*Myotis goudoti*	11	1
Anjohibe	Nov. 2014	Rhinonycteridae	*Paratriaenops furculus*	31	11
			Triaenops menamena	13	8
		Miniopteridae	*Miniopterus gleni*	2	1
			Miniopterus griveaudi	26	15
		Molossidae	*Otomops madagascariensis*	6	0
		Pteropodidae	*Rousettus madagascariensis*	41	2
		Vespertilionidae	*Myotis goudoti*	3	2
			Pipistrellus hesperidus	5	0
